# Exploring the Activation Mechanism of the mGlu5 Transmembrane Domain

**DOI:** 10.3389/fmolb.2020.00038

**Published:** 2020-03-06

**Authors:** Isaias Lans, Óscar Díaz, James A. R. Dalton, Jesús Giraldo

**Affiliations:** ^1^Laboratory of Molecular Neuropharmacology and Bioinformatics, Unitat de Bioestadística and Institut de Neurociències, Universitat Autònoma de Barcelona, Bellaterra, Spain; ^2^Biophysics of Tropical Diseases, Max Planck Tandem Group, University of Antioquia, Medellín, Colombia; ^3^Unitat de Neurociència Traslacional, Parc Taulí Hospital Universitari, Institut d’Investigació i Innovació Parc Taulí (I3PT), Institut de Neurociències, Universitat Autònoma de Barcelona, Barcelona, Spain; ^4^Instituto de Salud Carlos III, Centro de Investigación Biomédica en Red de Salud Mental, CIBERSAM, Madrid, Spain

**Keywords:** G protein-coupled receptors, mGlu5 receptor, molecular dynamics computer simulation, potential of mean force calculations, free energies, mGlu, class C GPCR

## Abstract

As a class C GPCR and regulator of synaptic activity, mGlu5 is an attractive drug target, potentially offering treatment for several neurologic and psychiatric disorders. As little is known about the activation mechanism of mGlu5 at a structural level, potential of mean force calculations linked to molecular dynamics simulations were performed on the mGlu5 transmembrane domain crystal structure to explore various internal mechanisms responsible for its activation. Our results suggest that the hydrophilic interactions between intracellular loop 1 and the intracellular side of TM6 have to be disrupted to reach a theoretically active-like conformation. In addition, interactions between residues that are key for mGlu5 activation (Tyr659^3.44^ and Ile751^5.51^) and mGlu5 inactivation (Tyr659^3.44^ and Ser809^7.39^) have been identified. Inasmuch as mGlu5 receptor signaling is poorly understood, potentially showing a complex network of micro-switches and subtle structure-activity relationships, the present study represents a step forward in the understanding of mGlu5 transmembrane domain activation.

## Introduction

Metabotropic glutamate (mGlu) receptors belong to Class C G protein-coupled receptors (GPCRs). mGlu receptors are composed of 8 subtypes assembled into 3 (I, II, and III) groups (Pin et al., 2003). The focus of the present study, mGlu receptor subtype 5 (mGlu5), belongs to Group I (Pin and Bettler, 2016). mGlu5 is involved in several neurologic and psychiatric disorders (Foster and Conn, 2017). Inhibition of mGlu5 might potentially alleviate depression, Parkinson’s disease, Fragile X syndrome and chronic pain symptoms whereas positive modulation of mGlu5 may provide a new treatment for schizophrenia (Nicoletti et al., 2015).

From a structural point of view, mGlu receptors are characterized by a dimeric arrangement of three protein domains: the extracellular Venus flytrap (VFT) domain, the transmembrane (TM) domain, and the cysteine rich domain (CRD). The VFT domain includes the orthosteric site where glutamate and synthetic agonists and antagonists bind. The TM domain includes the seven helices typical of all GPCRs and the intracellular regions responsible for G protein recognition. The CRD connects the VFT and TM domains (Pin and Bettler, 2016) mGlu receptors are obligate dimers, which adds both versatility and complexity to signal transduction (Rovira et al., 2008; Zhou and Giraldo, 2018; Pin et al., 2019).

In contrast to Class A GPCRs, the TM domains of mGlu receptors bear allosteric binding sites exclusively (Bennett et al., 2014). Ligand binding to the TM domain allows for positive, negative and silent allosteric modulation of glutamate activity (PAM, NAM, and SAM, respectively) (Kenakin, 2005; Conn et al., 2009). NAM can be either full or partial depending on whether full occupancy at the allosteric site provides total or partial target inhibition, respectively. Allosteric modulation may affect both the affinity and the efficacy of agonists. This can be done either in a convergent or a divergent way, as it has been recently shown (Kenakin and Strachan, 2018). As an example of the latter behavior, PAM-antagonists were defined as allosteric modulators that increase the affinity but decrease the efficacy of agonists (Kenakin and Strachan, 2018). Moreover, mGlu PAMs may exert a functional effect by themselves because their binding to the TM domain may directly activate the receptors, in particular mGlu5 receptor (Noetzel et al., 2012). This property divides mGlu PAMs into two groups: pure and ago-PAMs. The former group comprises those compounds that lack intrinsic efficacy and are not able to activate the receptor in the absence of glutamate or any other orthosteric agonist. Their mechanism of action is a pure allosteric enhancement of agonist activity. The latter group comprises those compounds that have both agonist and allosteric function (Foster and Conn, 2017). This different mechanism of action of mGlu PAMs may have therapeutic consequences. It has been shown that, in contrast to pure mGlu5 PAMs, mGlu5 ago-PAMs may present severe side effects such as induction of seizures and behavioral convulsions (Rook et al., 2013). This indicates that mGlu5 ligand recognition is complex and interpreted by at least two (agonist- and allosteric-) chemical languages that share many grammatical features.

Significant efforts have been made to decipher the mGlu5 receptor structure-activity translation machinery (Kaae et al., 2012; Dalton et al., 2014, 2017; Gregory et al., 2014; Gomez-Santacana et al., 2017; Cong et al., 2019; Jojart et al., 2019; Llinas Del Torrent et al., 2019). In general, a delicate molecular-gear receptor system whose mechanism of action is not yet well understood has become apparent. In this regard, it is remarkable that very small changes in ligand structure can have large effects in ligand function, transforming a PAM into a NAM or vice versa (Wood et al., 2011; Gomez-Santacana et al., 2014). Apparently, multiple triggers for agonism and allosterism coexist in the mGlu5 TM domain and work in a concerted and variable fashion. This finding seems not to be rare because as reviewed in Dosa and Amin (2016), functional switches appears to be a broad phenomenon reflecting the dynamic nature of GPCRs.

Of note, six key residues have been identified as part of the affinity receptor network in a rat mGlu5 receptor (Gregory et al., 2014). These residues can be translated to a human receptor sequence corresponding to Pro655^3.40^, Tyr659^3.44^, Thr781^6.46^, Trp785^6.50^, Ser809^7.39^, and Ala810^7.40^ [superscript numbering according to Pin et al in class C GPCRs (Pin et al., 2003), adapted from the Ballesteros-Weinstein scheme in Class A GPCRs (Ballesteros and Weinstein, 1995)]. Interestingly, the Trp785^6.50^Ala mutation has different effects depending on ligand structure: while a slight increase or decrease in co-operativity was observed for some PAM and NAM scaffolds, respectively, a NAM to PAM switch was detected for two different ligand scaffolds (Gregory et al., 2014). Also, it was found that Thr781^6.46^Ala and Ser809^7.39^Ala mutations switched the pharmacology of some alkyne type PAMs (Gregory et al., 2013). Thus, the receptor machine performs differently depending on the molecular program the allosteric modulator selects.

The recent determination of the crystal structures of the TM domain of the mGlu5 receptor, in an inactive state and bound to NAMs (Dore et al., 2014; Christopher et al., 2015) has definitively established the allosteric modulator binding region. This structural knowledge has confirmed the role of these and other residues previously identified by mutagenesis studies (Pagano et al., 2000; Malherbe et al., 2003, 2006; Gregory et al., 2013). More recently, a study was presented in which the inactive and active conformations of full-length mGlu5 dimer were elucidated by a combination of X-ray crystallography, cryo-electron microscopy and signaling studies (Koehl et al., 2019). As part of the mechanism of receptor activation, this work determined the intersubunit TM conformational change leading to a TM6-TM6 interface, as already proposed in a previous study (Hlavackova et al., 2012). In addition, the relevance of the ECL2 in the propagation of structural changes from the VFT to the TM through the CRD domain was pointed out (Koehl et al., 2019). However, the intrasubunit rearrangement which, accordingly to REFs (Hlavackova et al., 2012; Grushevskyi et al., 2019), should sequentially occur after the intersubunit conformational change was not identified, probably because of the absence of the G protein in the agonist-receptor complex. Nevertheless, though the structural characteristics of each of the protomers in the active TM domain remain unsolved, some features, which potentially could lead to TM activation, were found. In particular, an upward movement of TM3, a slight outward movement of TM5 and a destabilization of the ionic lock (Koehl et al., 2019).

Because of the complexity of mGlu molecular functioning, a complete solution to the problem can only be obtained by collecting different pieces of knowledge from complementary techniques. While analyses of crystallographic structures provide precise comparisons between static snapshots (Dalton et al., 2015; Lans et al., 2015a) molecular dynamics (MD) simulations supply the time dimension to the problem allowing the receptor to make use of its flexibility, thereby making visible dynamic interactions between particular receptor residues and revealing conformational effects (Latorraca et al., 2017).

To take a step forward in previous MD simulations of the mGlu5 receptor (Dalton et al., 2016, 2017) we provide herein potential of mean force (PMF) calculations (using umbrella sampling) linked to MD simulations of the TM domain of this receptor in its apo form. The present computational study, although conceived under a reductionist approach because it includes only one TM domain of a dimeric 3-domain receptor, has allowed the identification of some conformational features that can help to understand the intricacies of mGlu activation mechanism. The approach is consistent with experimental data that showed that the TM domain of a truncated mGlu5 receptor displays the same agonist-independent constitutive activity as the wild-type receptor (Goudet et al., 2004). Thus, the present study aims to structurally explore the mGlu5 TM domain constitutive activity.

## Results and Discussion

### MD Simulations of Apo mGlu5 TM Domain

The present work focuses on revealing key structural details of the activation mechanism of apo mGlu5 TM domain at the atomic level. To this end, the reported crystallographic structure of the mGlu5 TM domain in complex with the NAM mavoglurant (PDB ID: 4OO9) (Dore et al., 2014) was used as a reference state. Thus, the receptor displays the typical structural features of an inactive state. In particular, the ionic lock between Lys665^3.50^ on TM helix 3 (TM3) and Glu770^6.35^ (TM6), which is characteristic of the inactive state of Class C GPCRs, (Pin and Bettler, 2016) is closed. Additionally, Asn767 on intracellular loop 3 (ICL3) makes hydrogen bond interactions with Ser612 on intracellular loop 1 (ICL1), which extends from residues Tyr604 to Ser614.

Because the crystallographic structure of the mavoglurant-mGlu5 complex is partially incomplete and it also includes some modifications of the receptor amino acid sequence, it was necessary to work with a complete model of the *wt* TM domain structure in the apo form. To this end, a model developed previously by our group was used (see section “Methods”). Then, to simulate the activation of the mGlu5 TM domain, the receptor model was equilibrated and, subsequently, an MD simulation of 380 ns was carried out. The RMSD of the backbone atoms through the MD simulation showed a stabilization of the TM domain (Supplementary Figures S1, S2). Briefly, the TM domain maintains its inactive state, which means that the Lys665^3.50^-Glu770^6.35^ ionic lock and the Asn767-Ser612 interactions are conserved along the course of the MD simulation. Because of this, we did not observe any cavity formation at the intracellular side of the TM domain, which is presumably necessary for Gq coupling, based on currently known Class A GPCR-G protein crystal structures (Weis and Kobilka, 2018).

**FIGURE 1:**
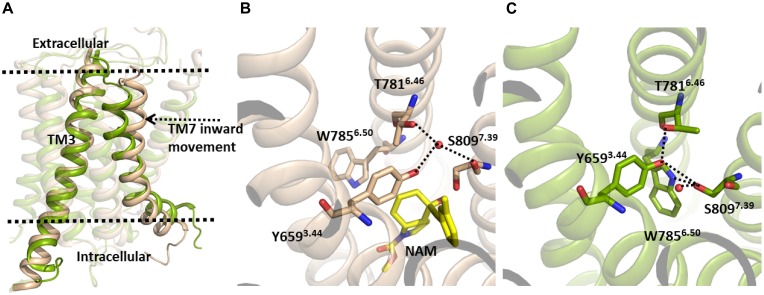
**(A)** The inactive TM domain of mGlu5. The initial structure is shown in brown and a snapshot after 380 ns of the MD simulation is shown in green. During the MD simulation, we observed an inward movement of TM7, with this helix approaching TM3. **(B)** Crystallographic structure of the allosteric site of the TM domain of the mGlu5-NAM complex (PDB ID: 4OO9) (Dore et al., 2014). **(C)** TM7 movement during the MD simulation promotes conformational changes of several side chains in the allosteric pocket. Tyr659^3.44^, Thr781^6.46^, Trp785^6.50^, and Ser809^7.39^ side chains rearrange to fill empty spaces inside the allosteric pocket yielding a narrowing of the allosteric site, which is maintained via a new hydrogen bonding network.

However, despite this, in our MD simulation of the mGlu5 TM domain, a number of structural changes were identified, with the main variations affecting the allosteric binding site (Figure 1). In particular, we observed a movement of the extracellular part of TM7 through which TM7 approaches TM3 (Figure 1, left). It is worth noting that our model was constructed from a crystal structure where the NAM mavoglurant is bound to the receptor in the allosteric binding site (Dore et al., 2014). However, in our apo model the allosteric binding site is empty. Thus, the space of the allosteric site was partially filled by TM7 during the MD simulation. As expected, the movement of TM7 affects some interactions in the allosteric site. In the mGlu5 crystal structure (Figure 1, center) there is a hydrogen bond network, mediated by a water molecule, involving Tyr659^3.44^ and Thr781^6.46^ side chains and the backbone carbonyl of Ser809^7.39^. In our simulation, Trp785^6.50^, Tyr659^3.44^ and Ser809^7.39^ side chains rearrange themselves in order to fill empty spaces inside the allosteric pocket. These movements are made in such a way that the side chain nitrogen of Trp785^6.50^, the hydroxyl group of Tyr659^3.44^, the hydroxyl group of Ser809^7.39^ and the Thr781^6.46^ side chain form direct hydrogen bonds between them rather than ones mediated by a water molecule. However, the hydroxyl group of Ser809^7.39^ is able to form a new hydrogen bond with a water molecule (Figure 1, right and Supplementary Figure S3). This collection of hydrogen bonds has, as a consequence, the narrowing of the allosteric site.

### mGlu5 TM Domain Activation Through Umbrella Sampling Simulations

**FIGURE 2:**
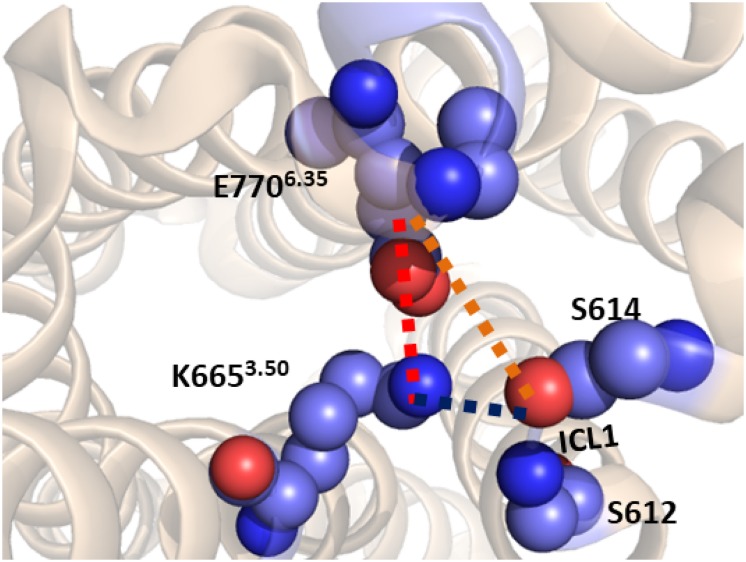
The three collective variables tested on the intracellular side of mGlu5 TM domain. (1) The center of mass distance between the Cα atoms of residues Ser612 and Ser614 (ICL1) and the Cα atoms of residues Asn767 (ICL3) and Glu770^6.35^ (TM6) (orange line). (2) The distance between the Lys665^3.50^-N*ξ* and Glu770^6.35^-Cδ (K665-N*ξ* and E770-Cδ) atoms (red line). (3) The center of mass distance between ICL1 and Lys665^3.50^-N*ξ* atom (ICL1 and K665-N*ξ*) (blue line).

Starting from the last receptor structure of the MD simulation described above, we calculated the PMF, using umbrella sampling simulation, for the activation of the TM domain of apo mGlu5. The PMF provides us with a free energy profile as a function of a collective variable, which can describe a specific process. Three collective variables were tested for modeling mGlu5 receptor activation: (1) the center of mass distance between the Cα atoms of residues Ser612 and Ser614 (both on ICL1) and the Cα atoms of residues Asn767 (ICL3) and Glu770^6.35^ (intracellular end of TM6). This distance represents the disruption/formation of an interaction at the intracellular side between TM6/ICL3 and ICL1. (2) The distance between the Lys665^3.50^-N*ξ* and Glu770^6.35^-Cδ atoms (K665-N*ξ* and E770-Cδ). This distance defines the ionic lock between TM3 and TM6 at the intracellular side. (3) The center of mass distance between ICL1 and atom Lys665^3.50^-N*ξ* (ICL1 and K665-N*ξ*). This distance represents the disruption/formation of an interaction between TM3 and ICL1 (specifically between K665 and S613) at the intracellular side of the receptor (Figure 2) as observed in the crystal structure (Dore et al., 2014).

The PMFs were computed at different simulation times (500 ps and 1, 3, and 4 ns). It was considered that the PMFs converged when the maximum change between the last PMF and the previous one was lower than 2 kcal/mol (Supplementary Figure S5). It can be seen that, in general, the histograms for the collective variables show a strong overlapping between adjacent windows. Such overlapping is a key point in umbrella sampling simulations. Two neighboring windows need to overlap with each other in order that a continuous PMF can be obtained from the calculations. Moreover, strong overlapping is required by the weighted histogram analysis method (WHAM) for PMF construction (Kästner, 2011). However, the parameter choice for umbrella sampling simulations (i.e., the spring constant and the number of umbrella sampling windows) is critical because greater overlapping requires more computational resources. Thus, a balance between overlapping and computation time is needed to reach a feasible PMF. In this regard, the ionic lock collective variable contained a considerable overlap in the present study. Consequently, in future studies, a better separation between adjacent windows would be necessary to ensure more efficient PMF computations.

**FIGURE 3:**
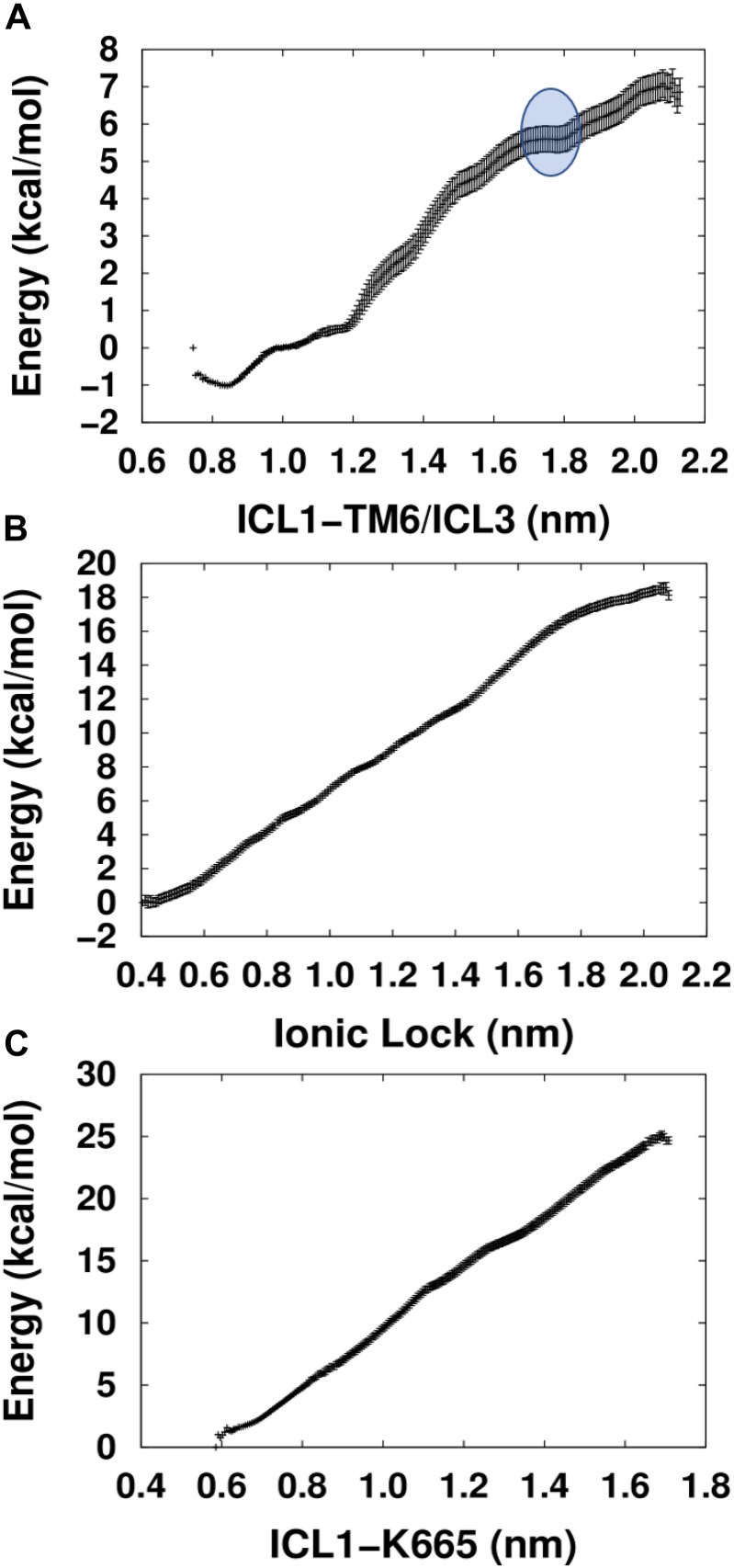
Calculated PMFs for the activation of the mGlu5 TM domain using three different collective variables. **(A)** The center of mass distance between ICL1 (Cα atoms of residues Ser612 and Ser614) and ICL3/TM6 (Cα atoms of residues Asn767 and Glu770^6.35^). **(B)** Distance between the ionic lock atoms Lys665^3.50^-N*ξ* and Glu770^6.35^-Cδ (K665-N*ξ*-E770-Cδ). **(C)** Distance between the center of mass of ICL1 and Lys665^3.50^-N*ξ* atom (ICL1 and K665-N*ξ*).

Comparison between the energetic profiles of the three PMFs used shows that the PMF calculated using the collective variable ICL1-TM6/ICL3 yielded the lowest energy values when attempting to activate the TM domain of mGlu5 (Figure 3). Interestingly, in contrast to the others, which display nearly linear relationships, the ICL1-TM6/ICL3 PMF shows an energetic profile that could reflect the exploration of structural features involved in the triggering of receptor activation. In particular, a region was detected (blue circle, top of Figure 3) which includes a critical point that appears to identify a higher-energy “pseudo-stable” receptor state. It could be hypothesized that this region corresponds to an active-like receptor conformation on the basis that separation of intracellular loops is predicted to be a necessary feature of mGlu receptor activation (Dalton et al., 2017). Interestingly, the PMF whose collective variable involves the explicit breaking of the Lys665-Glu770 ionic lock did not show any clear minimum along its trajectory. This is potentially in agreement with previous studies that showed that the breaking of the ionic lock is not a sufficient condition for GPCR activation (Dror et al., 2009; Lans et al., 2015b) because it is possible to find some inactive Class A GPCR structures with a broken ionic lock [see for instance A2AR PDB ID: 3EML (Jaakola et al., 2008) and β2AR PDB ID: 2RH1 (Cherezov et al., 2007)]. Furthermore, in MD simulations revealing ligand-mediated mGlu4 conformational change, it was found that receptor “activation” resulted in transient disruption of the ionic lock but not a clear break (Dalton et al., 2017).

We admit that the critical point found within the ICL1-TM6/ICL3 PMF is not a pronounced minimum and the structural and functional properties characteristic of a true minimum may not be given in this case. The reason for that may lay in the limitations of our approach, which has reduced the structural complexity of a three domain (VFT, CRD, and TM) mGlu dimer to a single TM domain. Moreover, the G protein, which would allow the intracellular stabilization of the active state, has not been included either. However, despite its structural simplicity, our approach may be useful enough for the structural exploration of constitutive mGlu5 activity. As such, we tentatively call the TM conformations included within the blue circle in Figure 3, “active-like conformations.”

**FIGURE 4:**
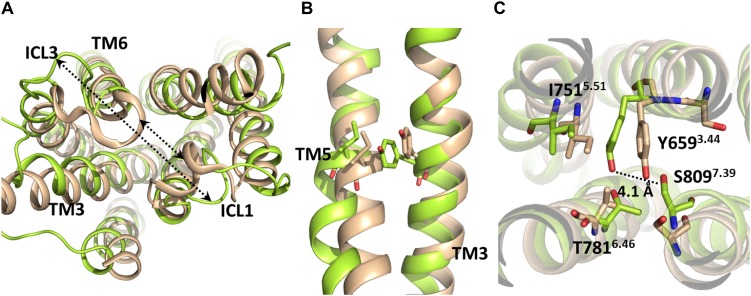
Molecular superposition between the inactive crystallographic (brown) and active-like (from-umbrella-sampling/MD, green) TM domains of mGlu5. **(A)** The arrows indicate the separation between ICL1-TM6/ICL3 after umbrella sampling simulation. **(B)** Lateral view of inactive and post-PMF/MD TM domains of mGlu5. Note the outward movement undergone by TM5 to reach an active-like conformation. **(C)** Bottom view of the inactive and post-PMF/MD TM domains of mGlu5. The dotted line shows the separation between Tyr659^3.44^ and Ser809^7.39^.

Due to the absence of G protein-bound crystal structures of class C GPCRs, the activation mechanism of mGlu receptors has been proposed as homologous to class A GPCRs. However, it should be noticed that class A and class C GPCRs share low sequence similarity (Pin et al., 2003) and, more noticeably, the micro-switches that have been linked to class A GPCR activation are not conserved in class C GPCRs. This includes sequence motifs in TM3 (DRY), TM6 (CWxP except for conserved Trp^6.48*a*6.50*c*^), TM7 (NPxxY) and the Ile^3.40*a*^, Pro^5.50*a*^, and Phe^6.44*a*^ residues of the “transmission switch” of class A GPCRs (Tehan et al., 2014) (superscripts numbering followed by “a” according to Ballesteros-Weinstein scheme (Ballesteros and Weinstein, 1995)] for class A GPCRs or followed by “c” according to Pin et al. (2003) scheme for class C GPCRs). Despite previous studies have proposed functional similarities between class A GPCR micro-switches and certain non-homologous class C GPCR residues (Perez-Benito et al., 2017; Llinas Del Torrent et al., 2019) their respective conformational changes have been observed only in MD simulations of mGlu receptor homology models, but not in mGlu receptor crystal structures (Dore et al., 2014; Christopher et al., 2015; Koehl et al., 2019) or in MD simulations starting from those (Feng et al., 2015; Dalton et al., 2017; Perez-Benito et al., 2017; Llinas Del Torrent et al., 2019). Furthermore, while the outward movement of TM6 is a hallmark of class A GPCR activation, the extent of this movement is noticeably lower in mGlu5 even when enhanced sampling methods are employed (Cong et al., 2019). Thus, the analogy between class A and class C GPCR conformational change and residue function is still uncertain, as no class C GPCR has been crystallized into a G protein-bound state up to date. The present study has identified some structural features that may distinguish the mGlu5 TM activation from that of class A GPCRs. Our simulations suggest that the most relevant interactions for the stabilization of the inactive state are those between TM6 and ICL1. The path from inactive to “active-like conformation” includes the reorientation of the flexible ICL3 (Figure 4 and Supplementary Figure S4) (an outward movement) and the breaking of the first H-bond of TM6: O(Glu770)-N(Ile774), which could be associated to the ICL3 outward movement. These changes were made without relevant movements or changes in TM6. Nevertheless, we have not found evidence indicating that the O(Glu770)-N(Ile774) H-bond needs to break for the receptor activation. Moreover, our results show that the cavity formation for the G-protein coupling (see section”Docking”) only requires slight movements of TM6. Thus, our results suggest a different activation mechanism of the TM domain of mGlu5 with respect to Class A GPCRs. Activation of Class A GPCRs requires the breaking of the ionic lock between TM3 and TM6 at their intracellular side and a large outward movement of TM6. However, it appears that the activation of the Class C GPCR mGlu5 requires, in addition to disruption of the ionic lock (Supplementary Figure S6), the breaking of interactions between the intracellular end of TM6 and ICL3 and ICL1. These changes can be made without affecting the general structure and stability of the receptor (Supplementary Figure S7 and Supplementary Movie S1). Furthermore, the present study suggests that the activation of the TM domain of mGlu5 does not involve an explicit outward movement or lengthening of TM6. Instead, TM5 and ICL3 may display a slight outward movement (Figure 4), which is a consequence of the ICL3-ICL1 separation and the very short ICL3 at mGlu5. This is further supported by the remarkably shorter length of the intracellular ends of TM5 and TM6 in mGlu receptors compared to class A GPCRs (i.e., TM5 and TM6 of β_2_ adrenergic receptor extend for 18 and 9 residues more than mGlu5 into the intracellular region, respectively, see section “Docking”). This data is in agreement with the importance of the conformation of intracellular loops for mGlu receptor activation and G protein binding found both experimentally (Gomeza et al., 1996; Francesconi and Duvoisin, 1998) and in MD simulations (Dalton et al., 2017).

The hypothesis proposed in this study for the mGlu5 TM domain activation is compatible with experimental studies. The TM domains linked to the active and inactive VFT states of the full-length mGlu5 obtained from cryo-EM did not significantly differ between them (Koehl et al., 2019). Importantly, the outward movement of TM6, observed in the activation of class A GPCRs, was not observed in active mGlu5 (Koehl et al., 2019). In addition, both active and inactive structures show a dimeric arrangement, with the two TM domains completely separated in the inactive VFT state. However, in the active VFT state the TM domains show a strong interaction between their TM6s, which appears to be a hallmark of the mGlu activation process (Koehl et al., 2019). Thus, it is reasonable to hypothesize that if the TM6-TM6 mutual stabilization is strong enough, the intrasubunit outward movement of TM6 is less likely. Alternatively, it has also been suggested that there is enough space for the intracellular part of the TM6 of at least one of the subunits to move outward (Pin et al., 2019). Finally, it is worth mentioning that a slight outward movement of TM5 can be observed in the VFT-active cryo-EM structure (Koehl et al., 2019) which if it could be associated to TM activation it would be consistent with the proposal presented in the present study.

**FIGURE 5:**
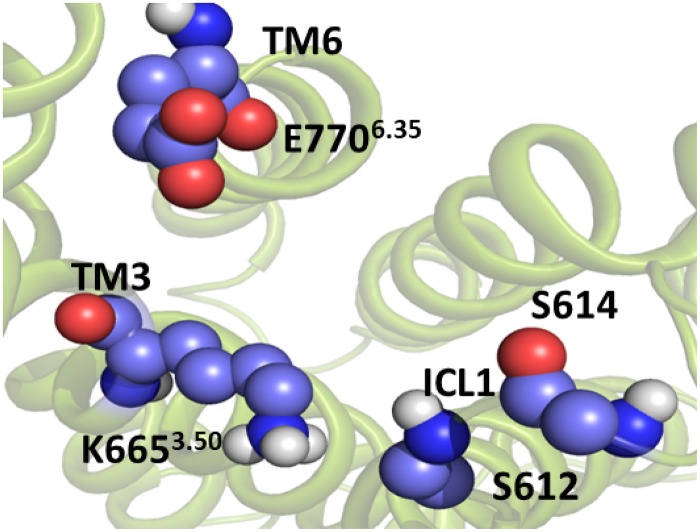
The intracellular active-like conformation of the TM domain of mGlu5. The structure was obtained by umbrella sampling/molecular dynamics simulation for the collective variable ICL1-TM6/ICL3. The interaction ASN767_Glu770^6.35^-ICL1 is disrupted. Because of this, there is a cavity formation in the intracellular side of the TM domain, which is likely to be necessary for Gq protein coupling.

In the active-like conformations identified in the present study by umbrella sampling simulation and through the unbiased trajectory of the MD simulation of the initially inactive mGlu5, we observed some residues in the allosteric binding site that undergo re-packing because of the deletion of the co-crystallized NAM (Figure 1). In order to detect conformational changes in the allosteric site that could be involved in the activation of the TM domain of mGlu5, we sought to identify similarities and differences between the inactive crystal state and active-like conformations of the receptor obtained from PMF application. We observed that Tyr659^3.44^ undergoes a conformational change in the active-like conformation with respect to the inactive one. As a consequence of this change, the aromatic ring of Tyr659^3.44^ is showed more displaced toward Ile751^5.51^ and the hydrogen bond between Tyr659^3.44^ and Ser809^7.39^ side chains is disrupted, showing a longer distance (Figure 4 and Supplementary Figure S3). All these structural features could be a consequence of the outward movement of TM5 through the activation-like process (Figure 4). However, this chain of events could occur in the opposite way. Thus, we speculate that having a bound ago-PAM, this could modulate Tyr659^3.44^ conformation on TM3 in a way that Tyr659^3.44^ is moved toward Ile751^5.51^ on TM5, thus forcing the outward movement of TM5. This movement could induce a conformational change of Asn767 located on intracellular loop 3 (ICL3) and Glu770^6.35^ (intracellular end of TM6). Thus, Asn767 and Glu770^6.35^ could break their interactions with ICL1 allowing the formation of a cavity in the intracellular side, necessary for the receptor activation (Figure 5). Thus, as it happens in class A GPCRs, one of the molecular triggers for the activation of the TM domain of mGlu5 could be a structural change initiated at TM3 (Dalton et al., 2015; Lans et al., 2015a).

### Protein-Protein Docking of Gq With the Active-Like Conformation of the TM Domain of mGlu5

Most of what is known of GPCR activation at structural level has come from the determination of active and inactive Class A crystal structures (Weis and Kobilka, 2018). Structural analyses have shown that agonist binding at the upper part of the receptor is not sufficient to activate the receptor and that concomitant G protein binding at the cytoplasmic side of the transmembrane domain of receptors is necessary (Gregorio et al., 2017).

To evaluate to what extent the “active-like” conformation of the TM domain of mGlu5 is active in the classical sense, we performed a protein-protein docking to examine the capability of the receptor to accommodate a bound GTPase and the helical domain of Gq protein (PDB ID: 3AH8) at its intracellular side (Nishimura et al., 2010). If the resulting docking complex would resemble the crystallographic β_2_ adrenergic receptor-Gs protein complex (PDB ID: 3SN6) (Rasmussen et al., 2011) it would be reasonable to consider the mGlu5 conformation as active.

**FIGURE 6:**
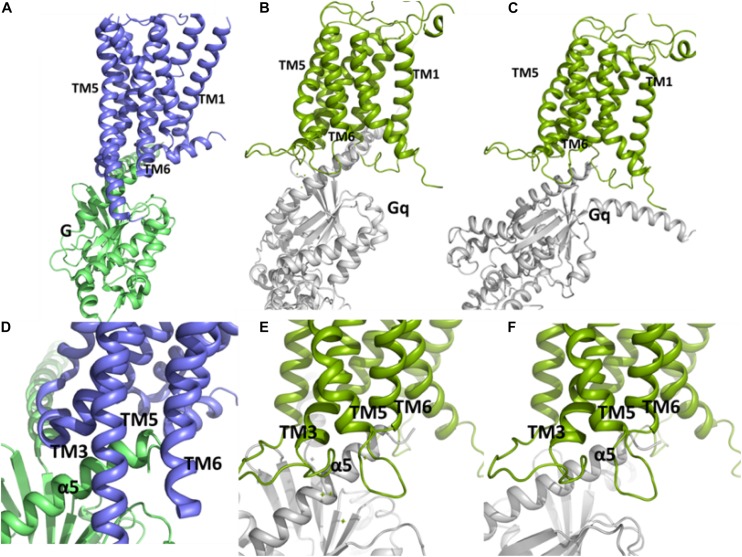
The conformations of the complex between Gq and the TM domain of mGlu5 obtained by using protein-protein docking using Cluspro **(B,E)** and Haddock **(C,F)** are similar to the crystallographic β_2_ adrenergic receptor-G protein complex (PDB ID: 3SN6) (Rasmussen et al., 2011) **(A,D)**. The C terminal of α5 helix within Gα subunit of Gq is located between the intracellular ends of TM3, TM5 and TM6.

We used Cluspro webserver (Kozakov et al., 2013) and HADDOCK2.2 webserver (van Zundert et al., 2016) to carry out the protein-protein docking. To validate the usefulness of Cluspro and Haddock to predict the correct interaction complex in this kind of system we carried out a docking of the Gs protein to the β_2_ adrenergic receptor. The closest pose of Gs, predicted from the docking to the crystal complex (3SN6) (Rasmussen et al., 2011) using Cluspro, belongs to the second populated cluster and has a RMSD of 7.2 Å with respect to the crystal structure (Supplementary Figure S8 and Supplementary Tables S1A,B). On the other hand, the best pose from Haddock has a RMSD of 2.41 Å with respect to the crystal structure. Thus, both servers are able to predict acceptable and medium quality poses of the complex Gs-β_2_ adrenergic receptor according to CAPRI (Critical Assessment of Prediction of Interactions) criterion (Lensink et al., 2007). Then, we performed a protein-protein docking between Gq and the “active-like” conformation of the TM domain of mGlu5 using Cluspro (Kozakov et al., 2013) and Haddock (van Zundert et al., 2016) web servers. The results show that our active-like receptor conformation is able to accommodate the binding of Gq at its intracellular side (Figure 6). In particular, two conformation of the Gq-mGlu5 complex, belonging to clusters 19 and 3, predicted using the balanced Cluspro scoring function and the Haddock score, respectively (Supplementary Tables S2A,B) resemble the crystal Gs-β_2_ adrenergic receptor-complex (PDB ID: 3SN6) (Rasmussen et al., 2011; Figure 6). Such as in the crystal complex, the C terminal of the α5 helix within the Gα subunit of the Gq protein fits between the intracellular ends of TM3, TM5 and TM6 (as well as in between ICL1, ICL2, and ICL3). Additionally, it is observed from Cluspro conformation, that Lys665^3.50^ (the positive counterpart of the ionic lock) forms a cation-π interaction with Tyr352 of the Gα subunit of Gq, showing a distance of 4.0 Å between the Cγ_Y351 and Nζ_K98 atoms (in the crystal Gs-β_2_ adrenergic receptor complex, the equivalent distance Cγ_Y391-Cζ_R131 is of 4.7 Å). Additionally, a docking between the mGlu5 TM inactive conformation and the Gq protein was carried out. The results indicate that the “active-like” conformation accommodates the Gq protein deeper in the intracellular part than the inactive conformation of the receptor does (Supplementary Figure S9). This is an indication that the receptor conformation obtained in the present study might represent an active conformation of the TM domain of mGlu5.

## Conclusion

The present study shows a plausible pathway for the activation of the TM domain of mGlu5. Our results indicate that polar interactions between ICL1 and the intracellular side of TM6 have to be disrupted to allow the cavity formation at the intracellular side of the transmembrane domain, which is likely to be necessary for Gq protein coupling. This finding is in agreement with a previous study (Dore et al., 2014) which reports that Ser613Ala mutation, in ICL1, and Glu770Ala mutation, in TM6, which presumably disrupt polar interactions between ICL1 and TM6, result in constitutive receptor activity. In this regard, the importance of ICL1 for recognizing G proteins in a class A GPCR has been recently shown by NMR experiments (Sounier et al., 2015). In addition, we have noted that the interaction between Tyr659^3.44^ and Ile751^5.51^ is maintained in the “active-like” mGlu5 conformation, indicating that this interaction might contribute to activation or positive modulation of mGlu5. Moreover, the interaction between Tyr659^3.44^ and Ser809^7.39^, mediated by a water molecule in the NAM-mGlu5 crystal structure or directly observed in our unbiased MD and missing in the active-like conformation, could serve to stabilize the inactive conformation. Indeed, previous studies (Gregory et al., 2013) showed that Tyr659Val, Thr781Ala, and Ser809Ala mutations induce changes in the pharmacology of several modulator compounds, changing these from PAMs to NAMs or SAMs.

Finally, we have shown that the active-like conformation of the TM domain of mGlu5 obtained in the present study is able to accommodate the Gq protein. It is worth noting that although we have not identified a clear minimum corresponding to the active-like conformation, the obtained conformation could represent a useful model of an active-like state. In this regard, it is likely that to obtain a clear minimum of the TM active state it would be necessary to simulate the activation process in presence of the extracellular VFT domain and as a dimeric receptor: a receptor model system beyond the scope of the present study.

## Experimental Procedures

Classical MD simulations were performed to study the activation of the TM domain of mGlu5 in the apo form. The GROMOS53A6 force field (Oostenbrink et al., 2004; Hess et al., 2008) was used. The initial structure used was a complete mGlu5 TM model, built in a previous study (Dalton et al., 2016) from the mavoglurant-mGlu5 crystal structure (PDB ID: 4OO9) (Dore et al., 2014) in which the NAM mavoglurant and all the co-crystallized water molecules were removed. In particular, the water molecule at the bottom of the allosteric binding pocket was removed in our mGlu5 apo model because in the crystal structure it displays molecular interactions with the receptor that are dependent on the NAM presence (Dore et al., 2014). Thus, a destabilization of the water molecule is expected when the NAM is absent. In agreement with this assumption, it was shown in a previous study (Dalton et al., 2017) that this water molecule is not stable in the MD simulations performed on the free mGlu5 receptor. We performed all simulations on a standard workstation using GROMACS v4.6.1 (Hess et al., 2008).

### Minimization

The ligand-free mGlu5 model was inserted into a pre-equilibrated and fully hydrated POPC lipid bilayer of 123 molecules (Supplementary Figure S1). Berger lipids parameters were used (Berger et al., 1997) (obtained from http://moose.bio.ucalgary.ca/). The embedding protocol was achieved using the inflateGRO methodology (Kandt et al., 2007). The water model used was the single point charge (SPC) model (Berendsen et al., 1981). To remove the positive net charge of the system, 14 Cl^–^ ions were added as counterions. The resulting systems were minimized up to the maximum force <1000.0 kJ.mol/nm using the steepest descent algorithm. During the minimization the positions of the protein and membrane atoms were restrained by using a force constant of 1000 kJ.mol^–1^.nm^–2^.

### Equilibration and MD Production

The equilibration process was carried out in three steps. The first step consisted of 100 ps of NVT simulation, in which the z-coordinate of the POPC phosphorous atoms in the membrane as well as the heavy atoms of the mGlu5 TM domain were restrained. The reference temperature was set at 310 K, using the modified Berendsen thermostat coupling method with a time constant of 0.1 ps. The second step consisted of 3 substeps. The first substep consisted of 3 ns of NPT simulation (restraining both the z-coordinate of the POPC phosphorous atoms in the membrane and the heavy atoms of the TM domain); a force constant of 10,000 kJ.mol^–1^.nm^–2^ for these atoms was used. In the second substep (2 ns length) only the movements of the heavy atoms of the TM domain were restrained by using a force constant of 10,000 kJ.mol^–1^.nm^–2^. In the third substep (3 ns length) only the movements of the heavy atoms of the TM domain were restrained by using a force constant of 1000 kJ.mol^–1^.nm^–2^. Finally, the third step consisted of 12 ns without any restraint. Then, an unbiased MD simulation production of 380 ns was carried out without imposing any condition. In each step, the reference temperature was set at 310 K, the Nosé-Hoover thermostat coupling method with a time constant of 1.0 ps was used, and the pressure was set at 1 atm by using a semi-isotropic method with a time constant of 5.0 ps. The particle mesh Ewald method (Essmann et al., 1995) was used to estimate the long range Coulomb interaction and periodic boundary conditions were used in all the steps.

### Potential of Mean Forces Calculation

The PMF was calculated to estimate the free energy profile of the activation of apo mGlu5 TM domain. The starting structure was the last snapshot of the previous MD production phase. Three different PMFs were determined using the umbrella sampling technique (Kumar et al., 1992; Boczko and Brooks, 1993; Rajamani et al., 2003) along three different collective variables (Figure 2): (1) The center of mass distance between ICL1 (Cαs of residues Ser612 and Ser614) and Cαs of residues Asn767 (ICL3) and Glu770^6.35^ (TM6) (orange line); (2) Distance between the K665^3.50^-N*ξ* and E770^6.35^-Cδ (K665-N*ξ* and E770-Cδ) atoms (red line); (3) the center of mass distance between ICL1 and Lys665^3.50^-N*ξ* atom (ICL1 and K665-N*ξ*) (blue line).

#### Umbrella Sampling Simulation

To choose the starting structures of the windows, we carried out 40 sequential MD simulations of 500 ps each, starting from the last snapshot of the unbiased apo MD simulation. The velocities and coordinates of the last configuration generated in each of the MD simulations were used as starting points for the following one and successively in this way to complete the 40 MD simulations. The sampling of each MD simulation was centered at a specific value of the collective variable (0.05 nm larger than the previous simulation) by using a spring constant of 4000 kJ/mol.nm^2^. Finally, from the set of 40 MD simulations, we localized 24 configurations whose values in the collective variable periodically increased 0.05 nm between them and, thus, let the receptor going from the inactive configuration to the active-like one. Each configuration was stabilized for at least 50 ps in each MD simulation. In this way, we started each umbrella sampling window from a stabilized structure. In the case of the ionic lock collective variable, it was necessary to localize 70 starting structures whose values in the collective variable periodically increased 0.017 nm between them and, thus, let the receptor going from the inactive configuration to the active-like one. This procedure allowed us to obtain a good sampling of the entire range of the collective variable. Subsequently, we carried out 4 ns MD per window for the ICL1-TM6/ICL3 and ICL1-K665 collective variables and 1 ns for the ionic lock. During the umbrella sampling simulations the trajectories were saved every 2 ps.

Finally, the PMFs were calculated using WHAM (Hub et al., 2010) implemented in GROMACS 4.6.1. Statistical errors were estimated using Bayesian bootstrap analysis (*N* = 50) (Hub et al., 2010).

### Protein-Protein Docking

The protein-protein docking was carried out using Cluspro webserver (Kozakov et al., 2013) with the balanced scoring function and Haddock 2.2 (van Zundert et al., 2016). To validate the usefulness of Cluspro and Haddock programs to predict the correct interaction complex in the GPCR-G protein system, we attempted to reproduce the Gs-β_2_ adrenergic X-RAY complex (PDB ID: 3SN6) (Rasmussen et al., 2011). Thus, we carried out a docking of the Gs protein to the β_2_ adrenergic receptor using the Gs and β_2_ adrenergic chains from the 3SN6 PDB structure (Rasmussen et al., 2011).

#### Cluspro Docking

The A chain (Thr9-Leu394) was used as ligand while the R chain (Glu30-Cys341) was used as receptor. To mimic the membrane or avoid poses of Gs protein interacting with the receptor part in contact with the membrane, we set some repulsive residues in the receptor (Glu30-Val48; Cys77-Glu122; Ile159-Tyr219 and Ile278-Gly320) and also in the ligand (Thr9-Gln19). Additionally, the Arg131 residue (in the receptor) and Arg389-Glu392 residues in the ligand were considered as attracting residues.

#### Haddock Docking

The A chain (Thr9-Leu394) was used as ligand while the R chain (Glu30-Cys341) was used as receptor. The Arg131 residue (in the receptor) and Arg389-Glu392 in the ligand were defined as active residues.

#### Gq-mGlu5 Docking

The GTPase and helical domains of Gq protein [PDB ID: 3AH8 (Nishimura et al., 2010) Gly1-Val355] were used as ligand while our “active-like” conformation of the TM domain of mGlu5 (obtained from the umbrella sampling simulation) was used as receptor. To mimic the membrane effect, in Cluspro web server, the residues Ser568-Val599, Leu622-Ser660, Val695-Phe756 and Tyr779-Glu815 (at the receptor) and Gly1-Met20 (at the ligand) were set as repulsive residues. On the contrary, Lys665 residue (positive counterpart in the ionic lock at mGlu5) and Leu349-Glu351 residues were set as attractive residues in the receptor and ligand, respectively. Finally, in Haddock web Server, Lys665 residue (positive counterpart in the ionic lock at mGlu5) and Leu349-Glu351 residues were set as active residues.

## Data Availability Statement

The datasets generated for this study are available on request to the corresponding author.

## Author Contributions

IL performed the MD simulations and PMF studies, analyzed the results, and wrote the manuscript. ÓD participated in structural analysis. JD revised the manuscript. JG supervised the study and wrote the manuscript.

## Conflict of Interest

The authors declare that the research was conducted in the absence of any commercial or financial relationships that could be construed as a potential conflict of interest.

## Supplementary Material

MOVIE S1 mGlu5 TM receptor model inserted in a POPC membrane model. Backbone RMSD of the mGlu5 TM domain relative to the starting structure of the unbiased molecular dynamics simulation. Time-window average (using a 10 ns window) of selected distances in the allosteric site along the unbiased molecular dynamics simulation. Backbone RMSD of the ICL3, relative to the starting structure of the unbiased molecular dynamics simulation. The PMF for each collective variable calculated at different simulation times. Average distance of the Ionic lock along the ICL1-TM6/ICL3 collective variable. RMSD of the receptor backbone with respect to the inactive structures as a function of the value of the ICL1-TM6/ICL3 collective variable. The conformation of the complex between Gs and the TM domain of β_2_ adrenergic receptor, obtained by protein-protein docking using Cluspro (blue-cyan complex) and Haddock2.2 (blue-yellow complex), are similar to the crystallographic β_2_ adrenergic receptor-G protein complex (PDB ID: 3SN6, blue-green complex) with a RMSD of 7.2 Å and 2.4 Å, respectively. Comparison of the inactive mGlu5 TM-Gq pose with the “active-like” mGlu5 TM-Gq pose. Cluspro docking rank between the Gs protein and the β_2_ adrenergic receptors. Haddock docking rank between the Gs protein and the β_2_ adrenergic receptors. Cluspro docking rank between the “active-like” conformation of the TM domain of mGlu5 and the GTPase and helical domain of Gq protein (3AH8 PDB). Haddock docking rank between the “active-like” conformation of the TM domain of mGlu5 and the GTPase and helical domain of Gq protein (PDB ID: 3AH8). Cluspro docking rank between the inactive conformation of the TM domain of mGlu5 and the GTPase and helical domain of Gq protein (PDB ID: 3AH8). Haddock docking rank between the inactive conformation of the TM domain of mGlu5 and the GTPase and helical domain of Gq protein (PDB ID: 3AH8).
