# Supplementary material for: Exploring the Activation Mechanism of the mGlu5 Transmembrane Domain
Source: Front Mol Biosci. 2020 Mar 6;7:38. doi: 10.3389/fmolb.2020.00038 (PMC7069277; doi:10.3389/fmolb.2020.00038)
Supplement: Supplementary file 2 [file Table_1.pdf]

Supporting Information

Exploring the activation mechanism of the mGlu5 transmembrane domain

Isaias Lans[1,4], Óscar Díaz[1,2,3] , James A. R. Dalton[1,2,3] and Jesús Giraldo[1,2,3]*

[1]Laboratory of Molecular Neuropharmacology and Bioinformatics, Unitat de Bioestadística and Institut de Neurociències, Universitat Autònoma de Barcelona, 08193 Bellaterra, Spain

[2]Unitat de Neurociència Traslacional, Parc Taulí Hospital Universitari, Institut d'Investigació i Innovació Parc Taulí (I3PT), Institut de Neurociències, Universitat Autònoma de Barcelona, Spain

[3]Instituto de Salud Carlos III, Centro de Investigación Biomédica en Red de Salud Mental, CIBERSAM, Spain

[4]Biophysics of Tropical Diseases, Max Planck Tandem Group, University of Antioquia, Medellín, Colombia

*Corresponding author:

Jesús Giraldo

Laboratory of Molecular Neuropharmacology and Bioinformatics, Unitat de Bioestadística and Institut de Neurociències, Universitat Autònoma de Barcelona. 08193 Bellaterra, Spain

Phone: +34 93 581 38 13; Fax : +34 93 581 23 44; Email : Jesus.Giraldo@uab.es

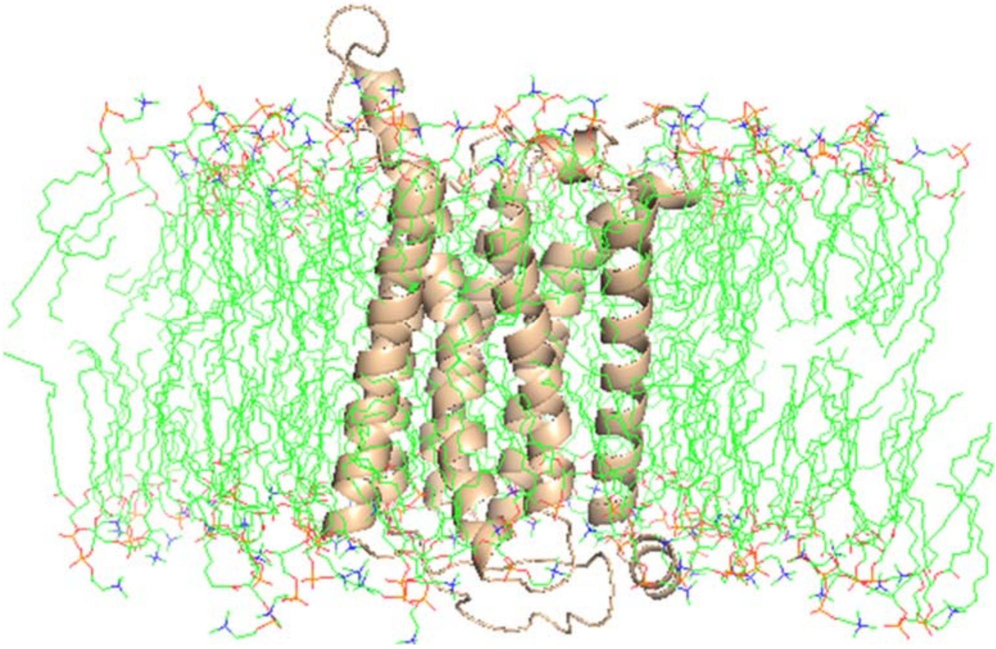

Figure SI1. mGlu5 TM receptor model inserted in a POPC membrane model.

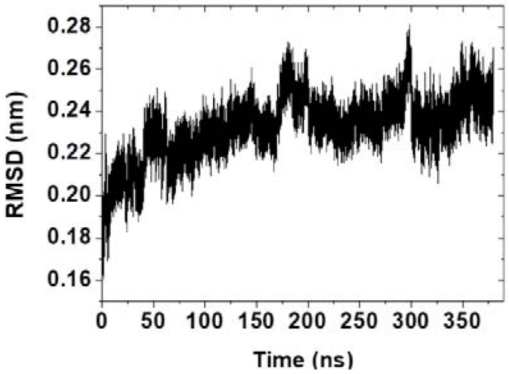

Figure SI2. Backbone RMSD of the mGlu5 TM domain relative to the starting structure of the unbiased molecular dynamics simulation.

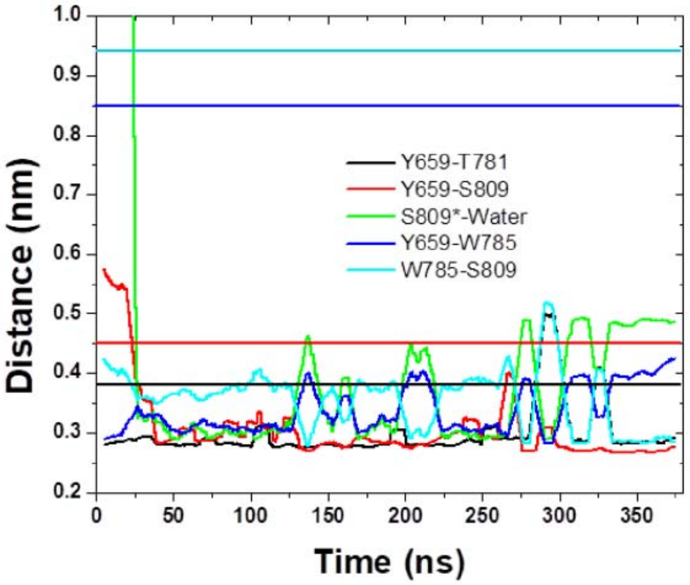

Figure SI3. Time-window average (using a 10 ns window) of selected distances in the allosteric site along the unbiased molecular dynamics simulation. The horizontal lines represent the corresponding distances observed at the crystallographic PDB ID: 4OO9 structure. Black, red, green, blue and cyan lines represent the OH_Y659-OH_T781, OH_Y659-OH_S809, OH_S809-O_Water, OH_ Y659-N_Side_chainW785 and N_Side_chainW785-OH_S809 distances, respectively.

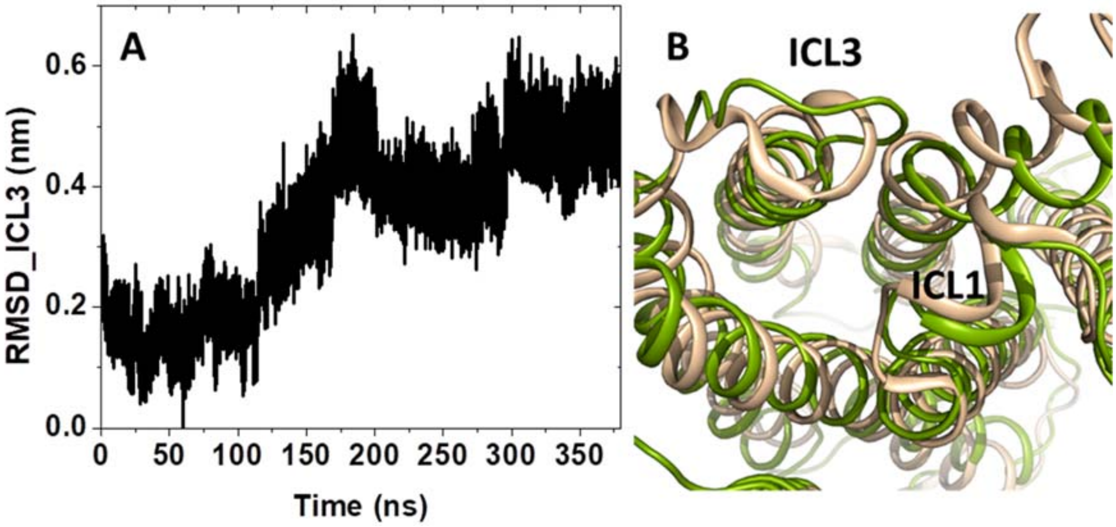

Figure SI4. Backbone RMSD of the ICL3 relative to the starting structure of the unbiased molecular dynamics simulation. The snapshot at 350 ns and the starting structure are showed in green and brown, respectively.

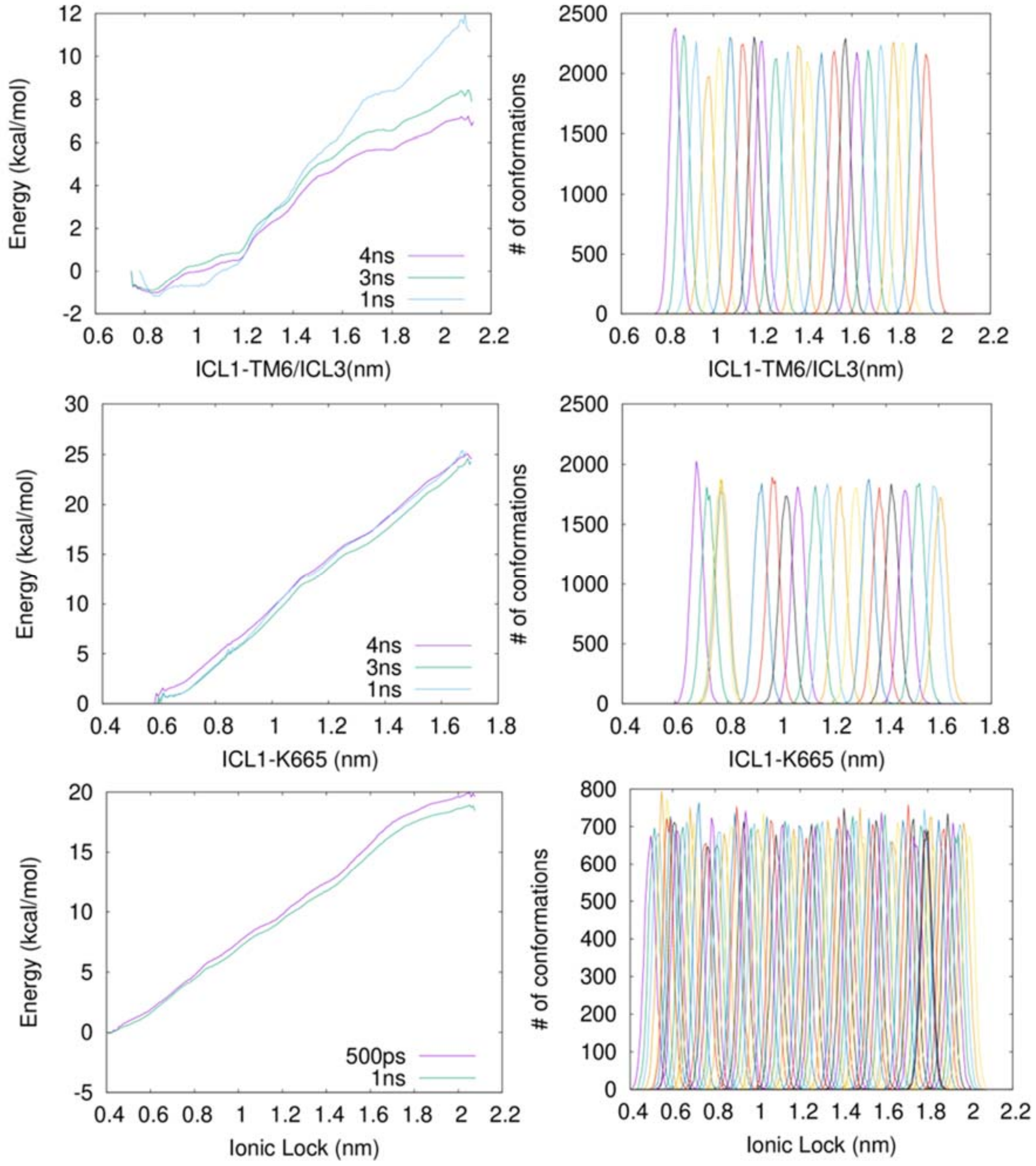

Figure SI5. The PMF for each collective variable was calculated at different simulation times. The plots on the left show the convergence of the PMFs (small changes in the PMF profile are observed between the last 2 PMFs for each collective variable). The plots on the right show the histograms for each collective variable. Although the PMFs for the ionic lock collective variable were calculated at 500 ps and 1 ns, the number of windows used was 3 times greater than the number of windows used for the ICL1-TM6/ICL3 and ICL1-K665 collective variables.

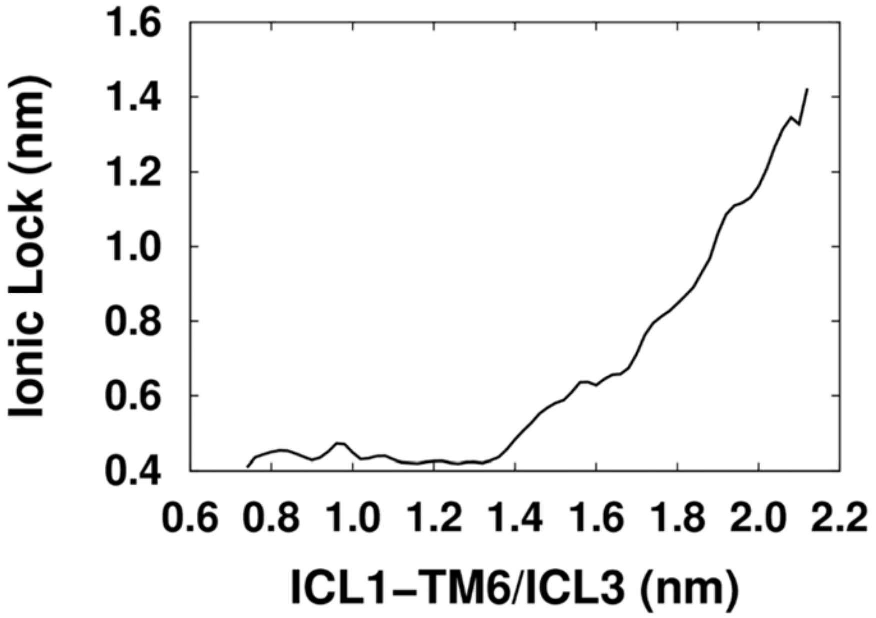

Figure SI6. Average distance of the Ionic lock along the ICL1-TM6/ICL3 collective variable.

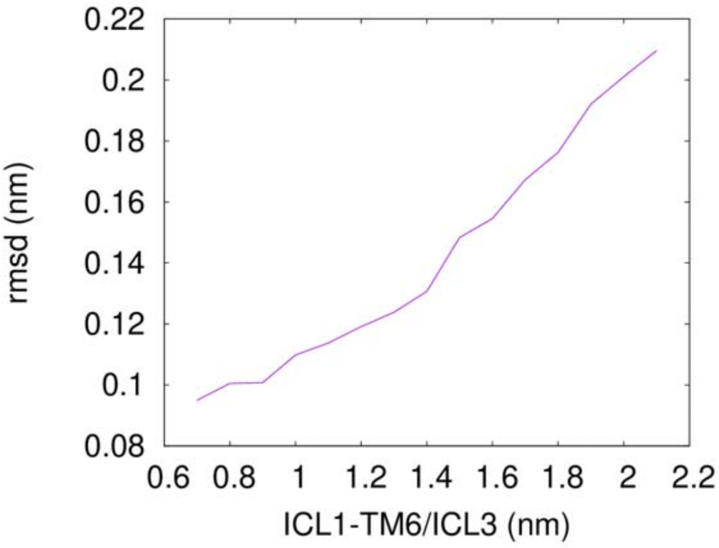

Figure SI7. RMSD of the receptor backbone with respect to the inactive structures as a function of the value of the ICL1-TM6/ICL3 collective variable. The folding of the mGlu5 TM domain does not show significant changes during the umbrella sampling simulation (compare with the unbiased molecular dynamics simulation, Figure SI2).

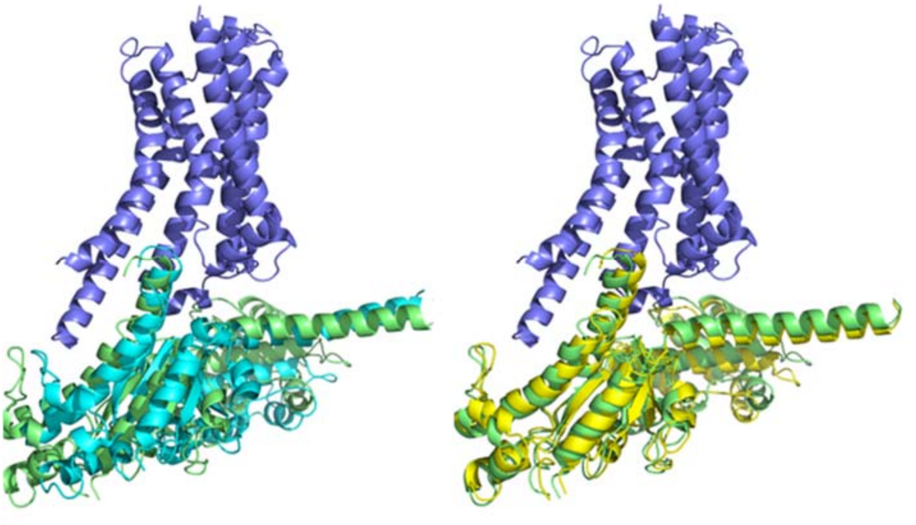

Figure SI8. The conformation of the complex between Gs and the TM domain of β_2 adrenergic receptor, obtained by protein-protein docking using Cluspro (blue-cyan complex) and Haddock2.2 (blue-yellow complex), are similar to the crystallographic β_2 adrenergic receptor-G protein complex (PDB ID: 3SN6, blue-green complex) with a RMSD of 7.2 Å and 2.4 Å, respectively.

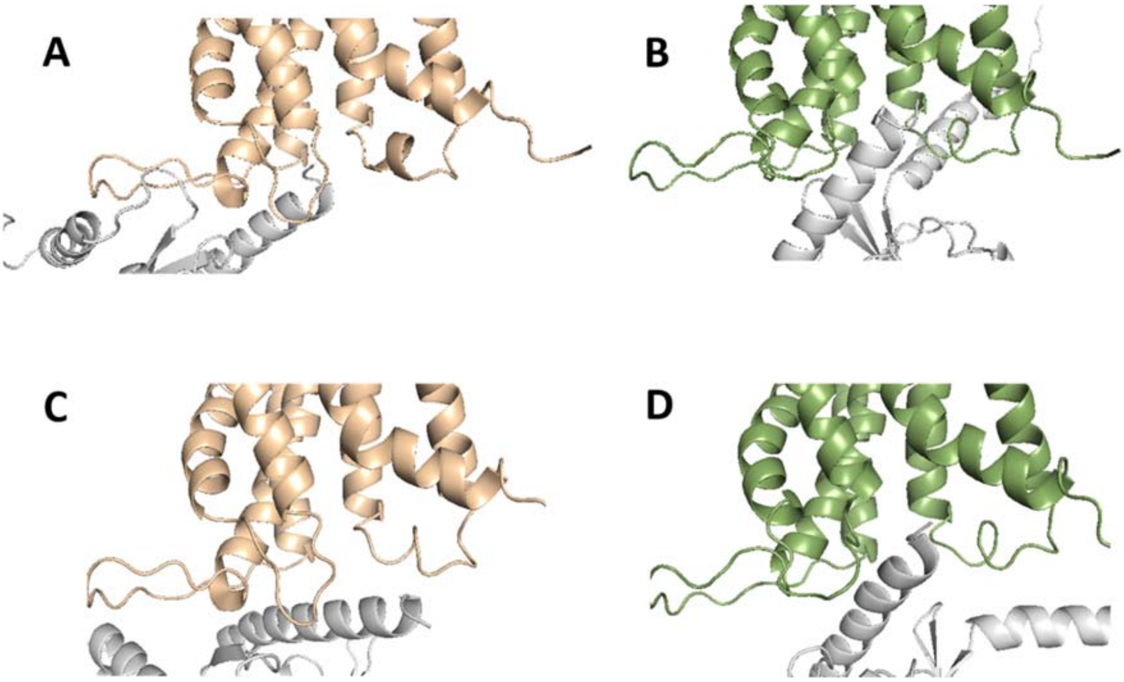

Figure SI9. Comparison of the inactive mGlu5 TM-Gq pose with the “active-like” mGlu5 TM-Gq pose. In the poses corresponding to the “active-like” mGlu5 TM conformation (green) and Gq protein (gray) obtained with Cluspro (B) and Haddock2.2 (D), the Gq penetrates into the intracellular part of the receptor. In the poses corresponding to the inactive mGlu5 TM conformation (brown) and Gq protein (gray) obtained with Cluspro (Cluster 2, Table SI3a) (A) and Haddock2.2 (Cluster 1 Table SI3b) (C) this penetration is not seen.

Table SI1a. Cluspro docking rank between the Gs protein and the β_2 adrenergic receptors. The coordinate of Gs protein and the β_2 adrenergic receptor were taken from the crystallographic complex with PDB ID: 3SN6.

Cluster	Members	Representative	Weighted Score
0	123	Center	-761.2
0	123	Lowest Energy	-1043.3
1	83	Center	-873
1	83	Lowest Energy	-873
2	57	Center	-704.3
2	57	Lowest Energy	-832.8
3	56	Center	-797.6
3	56	Lowest Energy	-810.1
4	46	Center	-800.6
4	46	Lowest Energy	-928.8
5	39	Center	-734.9
5	39	Lowest Energy	-910.8
6	36	Center	-716.5
6	36	Lowest Energy	-800
7	32	Center	-723.8
7	32	Lowest Energy	-876.2
8	31	Center	-732.3
8	31	Lowest Energy	-761.1
9	30	Center	-715.9
9	30	Lowest Energy	-918
10	30	Center	-779.8
10	30	Lowest Energy	-887.4
11	27	Center	-754.7
11	27	Lowest Energy	-822.2
12	24	Center	-759.6
12	24	Lowest Energy	-946.8
13	24	Center	-697.6
13	24	Lowest Energy	-771.3
14	23	Center	-689.8
14	23	Lowest Energy	-802.3
15	19	Center	-694.3
15	19	Lowest Energy	-761.7
16	18	Center	-711.1
16	18	Lowest Energy	-808.8
17	18	Center	-693
17	18	Lowest Energy	-802.5
18	18	Center	-709.4

18	18	Lowest Energy	-750
19	17	Center	-688.4
19	17	Lowest Energy	-757.7
20	15	Center	-764.9
20	15	Lowest Energy	-774.2
21	14	Center	-689.6
21	14	Lowest Energy	-863.7
22	14	Center	-695.8
22	14	Lowest Energy	-828.5
23	12	Center	-709.9
23	12	Lowest Energy	-737.6
24	11	Center	-692.4
24	11	Lowest Energy	-743.6
25	11	Center	-712.5
25	11	Lowest Energy	-744.1
26	11	Center	-735.5
26	11	Lowest Energy	-735.5
27	9	Center	-708.8
27	9	Lowest Energy	-724.8
28	9	Center	-720.1
28	9	Lowest Energy	-743.6
29	7	Center	-695.5
29	7	Lowest Energy	-756.4

Table SI1b. Haddock docking rank between the Gs protein and the β_2 adrenergic receptors. The coordinate of Gs protein and the β_2 adrenergic receptor were taken from the crystallographic complex with PDB ID: 3SN6.

Cluster	Members	Haddock score
12	4	-111.4
5	12	-99.5
1	31	-97.7
4	12	-81.5
2	25	-78.4
11	6	-77
9	7	-74
3	16	-66.2
8	7	-64.8
6	10	-63.6

Table SI2a. Cluspro docking rank between the "active-like" conformation of the TM domain of mGlu5 and the GTPase and helical domain of Gq protein (PDB ID: 3AH8).

Cluster	Members	Representative	Weighted Score
0	112	Center	-915.6
0	112	Lowest Energy	-1155.6
1	111	Center	-966.3
1	111	Lowest Energy	-1253.5
2	99	Center	-933
2	99	Lowest Energy	-1049.4
3	80	Center	-1058
3	80	Lowest Energy	-1225.8
4	64	Center	-889.5
4	64	Lowest Energy	-1046.7
5	52	Center	-894.4
5	52	Lowest Energy	-1278.2
6	50	Center	-1019.6
6	50	Lowest Energy	-1215.3
7	42	Center	-896.9
7	42	Lowest Energy	-1090.3
8	36	Center	-909.1
8	36	Lowest Energy	-1015.9
9	32	Center	-886.7
9	32	Lowest Energy	-1014
10	28	Center	-954.4
10	28	Lowest Energy	-954.4
11	27	Center	-916.1
11	27	Lowest Energy	-1054.8
12	26	Center	-1001.7
12	26	Lowest Energy	-1010.3
13	24	Center	-916.7
13	24	Lowest Energy	-973.6
14	24	Center	-1071.1
14	24	Lowest Energy	-1071.1
15	22	Center	-884.4
15	22	Lowest Energy	-960.3
16	20	Center	-869.7
16	20	Lowest Energy	-1177.4
17	20	Center	-897.8
17	20	Lowest Energy	-981.2
18	17	Center	-901.3
18	17	Lowest Energy	-990.3

19	15	Center	-887.2
19	15	Lowest Energy	-961.9
20	14	Center	-889.3
20	14	Lowest Energy	-917.9
21	13	Center	-872.8
21	13	Lowest Energy	-938.8
22	13	Center	-969.3
22	13	Lowest Energy	-969.3
23	12	Center	-872.7
23	12	Lowest Energy	-933.7
24	12	Center	-989
24	12	Lowest Energy	-1008.9
25	10	Center	-927.1
25	10	Lowest Energy	-939.1
26	10	Center	-907.3
26	10	Lowest Energy	-950.2
27	8	Center	-902.7
27	8	Lowest Energy	-930.2

Table SI2b. Haddock docking rank between the “active-like” conformation of the TM domain of mGlu5 and the GTPase and helical domain of Gq protein (PDB ID: 3AH8).

Cluster	Members	Haddock score
4	9	-105.1
2	32	-100.6
1	81	-97
8	4	-91.6
3	9	-86.5
5	8	-73.4
6	6	-59.2
7	4	-34.3

Table SI3a. Cluspro docking rank between the inactive conformation of the TM domain of mGlu5 and the GTPase and helical domain of Gq protein (PDB ID: 3AH8).

Cluster	Members	Representative	Weighted Score
0	103	Center	-708.3
		Lowest Energy	-808.5
1	70	Center	-701.4
		Lowest Energy	-861.8
2	50	Center	-826.5
		Lowest Energy	-831.4
3	43	Center	-706.8
		Lowest Energy	-847.6
4	42	Center	-659.1
		Lowest Energy	-760.9
5	40	Center	-714.6
		Lowest Energy	-781.0
6	38	Center	-684.0
		Lowest Energy	-729.6
7	33	Center	-672.4
		Lowest Energy	-768.2
8	33	Center	-681.3
		Lowest Energy	-796.6
9	31	Center	-655.8
		Lowest Energy	-854.2
10	30	Center	-777.8
		Lowest Energy	-777.8
11	29	Center	-705.3
		Lowest Energy	-789.5

12	24	Center	-667.1
		Lowest Energy	-707.0
13	23	Center	-690.9
		Lowest Energy	-783.3
14	22	Center	-716.2
		Lowest Energy	-720.9
15	22	Center	-698.8
		Lowest Energy	-736.0
16	22	Center	-769.9
		Lowest Energy	-769.9
17	21	Center	-652.0
		Lowest Energy	-737.1
18	18	Center	-664.2
		Lowest Energy	-765.0
19	17	Center	-657.8
		Lowest Energy	-771.1
20	16	Center	-741.0
		Lowest Energy	-741.0
21	15	Center	-711.7
		Lowest Energy	-711.7
22	14	Center	-690.3
		Lowest Energy	-731.7
23	14	Center	-757.6
		Lowest Energy	-757.6
24	13	Center	-834.1
		Lowest Energy	-834.1
25	12	Center	-749.0
		Lowest Energy	-749.0
26	12	Center	-692.2
		Lowest Energy	-692.2
27	10	Center	-672.0
		Lowest Energy	-690.9
28	8	Center	-653.4
		Lowest Energy	-733.8
29	5	Center	-658.5
		Lowest Energy	-691.7

Table SI3b. Haddock docking rank between the inactive conformation of the TM domain of mGlu5 and the GTPase and helical domain of Gq protein (PDB ID: 3AH8).

Cluster	Members	Haddock score
3	31	-110.6
1	68	-104.3
4	8	-102.7
6	7	-97.5
2	43	-93.4
5	7	-67.3
7	4	-67.3
8	4	-41.8
